# Supplementary material for: Understanding family-level decision-making when seeking access to acute surgical care for children: Protocol for a cross-sectional mixed methods study
Source: PLoS One. 2024 Jun 24;19(6):e0304165. doi: 10.1371/journal.pone.0304165 (PMC11195935; doi:10.1371/journal.pone.0304165)
Supplement: S1 File — (PDF) [file pone.0304165.s001.pdf]

SUPPLEMENTAL FILE 1

ADULT/CHILD DYAD SEMI-STRUCTURED INTERVIEW GUIDE

Introduction: Thank you for agreeing to participate in this interview. We are interviewing you to better understand how we can improve surgical care for children. There are no right or wrong answers to any of our questions – we are interested in your own experiences.

Participation in this study is voluntary and will not affect the care you currently receive. The interview should take approximately 45 minutes depending on how much information you would like to share. With your permission, I would like to video record the interview because I don’t want to miss any of your comments. All responses will be kept confidential. Do you have any questions before we begin?

May I turn on the digital recorder?

Theme	Corresponding Quant Data Source	ADULT Questions	Sub-question (s)	CHILD Questions
Experience seeking care		*Can you tell me about your experience with your child's appendicitis?*	(Limited history with timeline & decision making along the way)	
Interpreting signs & symptoms		*When did you first notice any signs or symptoms?*	*How did you interpret those symptoms?*<br>*How did you know that this was abnormal? What made you pay attention to this?*	*When did you first notice any signs or symptoms?*<br>*What did you think those symptoms meant?*<br>*How did you know that this was not normal? What made you pay attention to this?*<br>*Who did you talk to (i.e., go to your teacher)?*
Child/Parent Communication	ARCS	*Can you describe the communication between you and your child about his/her/their pain?*<br><br>*What made you recognize that you needed to seek care at the hospital?*	*Who initiated the conversation? Who pressed about it? How?*<br><br>*How did your child's description impact your decision making?*	*How did X (teacher, parent, etc.) respond when you told them about your X (abdominal pain)?*
Challenges at the point of accessing care	SILS	*What were the hard things about accessing care? (i.e., care coverage for other children, transportation, financial worries, distance from hospital, family, language challenges, etc.)*	*How did you pivot in your decision making because of X?*	

Strengths at the point of accessing care		*What helped when you were seeking care? (i.e., family support, social support, financial support)*		
Health Information – Person(s)		*Who did you turn to for help or advice (provider, family member, friend, etc.)? Why did you turn to this person?*<br><br>*Who do you trust most to get health information from – why?*		*Who did you turn to for help or advice (family member, school nurse, friend, etc.)? Why did you turn to this person?*
Health Information- Platform(s)		*What sources of health information did you use to understand your child's condition? (i.e., websites, social media) Why and why not other sources?*	*If so, how useful, or not useful, were they in helping you understand and make decisions?*	*Did you use the internet or social media to understand what you were feeling? If so, how did you find it useful?*
Self-identity		*Did you perceive any self-identifying factors that influenced your decision making during this experience?*		Repeat adult questions for children >12 y/o.<br><br>*What do you think?*

Conclusion

Is there anything else that you would like to share?
