# Supplementary material for: Understanding family-level decision-making when seeking access to acute surgical care for children: Protocol for a cross-sectional mixed methods study
Source: PLoS One. 2024 Jun 24;19(6):e0304165. doi: 10.1371/journal.pone.0304165 (PMC11195935; doi:10.1371/journal.pone.0304165)
Supplement: Attachment — Submitted filename: Response to Reviewers.docx [file pone.0304165.s002.docx]

Journal Requirements:

When submitting your revision, we need you to address these additional requirements.

1. Please ensure that your manuscript meets PLOS ONE's style requirements, including those for file naming. The PLOS ONE style templates can be found at 
[https://journals.plos.org/plosone/s/file?id=wjVg/PLOSOne_formatting_sample_main_body.pdf](https://nam04.safelinks.protection.outlook.com/?url=https%3A%2F%2Furldefense.com%2Fv3%2F__https%3A%2F%2Fjournals.plos.org%2Fplosone%2Fs%2Ffile%3Fid%3DwjVg*PLOSOne_formatting_sample_main_body.pdf__%3BLw!!OToaGQ!rQMlusJBBcqeJ1PSZVlq_mjiSDK4aFCHvyNOOyQ2usv1fMmOqHfFR1Wej2IaoonN_etN33FjDv6KSYXiVw%24&data=05%7C02%7Cbriajh%40vt.edu%7C41f5a54f31e2498b9f9b08dc3ca2d99a%7C6095688410ad40fa863d4f32c1e3a37a%7C0%7C0%7C638451917477437476%7CUnknown%7CTWFpbGZsb3d8eyJWIjoiMC4wLjAwMDAiLCJQIjoiV2luMzIiLCJBTiI6Ik1haWwiLCJXVCI6Mn0%3D%7C0%7C%7C%7C&sdata=xwOZPthpXjTRfmscvHIaTq5kbSnLCdjo5qisVsKZBiw%3D&reserved=0) and 
[https://journals.plos.org/plosone/s/file?id=ba62/PLOSOne_formatting_sample_title_authors_affiliations.pdf](https://nam04.safelinks.protection.outlook.com/?url=https%3A%2F%2Furldefense.com%2Fv3%2F__https%3A%2F%2Fjournals.plos.org%2Fplosone%2Fs%2Ffile%3Fid%3Dba62*PLOSOne_formatting_sample_title_authors_affiliations.pdf__%3BLw!!OToaGQ!rQMlusJBBcqeJ1PSZVlq_mjiSDK4aFCHvyNOOyQ2usv1fMmOqHfFR1Wej2IaoonN_etN33FjDv54qoKaXQ%24&data=05%7C02%7Cbriajh%40vt.edu%7C41f5a54f31e2498b9f9b08dc3ca2d99a%7C6095688410ad40fa863d4f32c1e3a37a%7C0%7C0%7C638451917477443287%7CUnknown%7CTWFpbGZsb3d8eyJWIjoiMC4wLjAwMDAiLCJQIjoiV2luMzIiLCJBTiI6Ik1haWwiLCJXVCI6Mn0%3D%7C0%7C%7C%7C&sdata=mYK7dyn5DgsoMxbgNe4cyxbvSHF5d0pxjs5N3WAqTIw%3D&reserved=0)

Manuscript format has been corrected to meet style requirements.

2. Please note that funding information should not appear in any section or other areas of your manuscript. We will only publish funding information present in the Funding Statement section of the online submission form. Please remove any funding-related text from the manuscript.

Thank you for this note. This has been amended.

3. Thank you for stating the following financial disclosure: 
   "BH and MR received the standard graduate student funding from Virginia Tech Carilion School of Medicine to support subject compensation for this study (see funding letter for reference)."

Please state what role the funders took in the study.  If the funders had no role, please state: "The funders had no role in study design, data collection and analysis, decision to publish, or preparation of the manuscript." 
If this statement is not correct you must amend it as needed. 
Please include this amended Role of Funder statement in your cover letter; we will change the online submission form on your behalf.

The funders had no role in this study, and as advised, the amended Role of Funder statement is now included in our cover letter.

4. Your ethics statement should only appear in the Methods section of your manuscript. If your ethics statement is written in any section besides the Methods, please move it to the Methods section and delete it from any other section. Please ensure that your ethics statement is included in your manuscript, as the ethics statement entered into the online submission form will not be published alongside your manuscript.

Corrected. Ethics statement moved to the methods section of manuscript.

5. Please review your reference list to ensure that it is complete and correct. If you have cited papers that have been retracted, please include the rationale for doing so in the manuscript text, or remove these references and replace them with relevant current references. Any changes to the reference list should be mentioned in the rebuttal letter that accompanies your revised manuscript. If you need to cite a retracted article, indicate the article’s retracted status in the References list and also include a citation and full reference for the retraction notice.

Reference list has been revised for completeness and proper formatting. Changes noted below:

**References added:**

Reference for race and ethnicity definitions: US Department of Health and Human Services O of MH. Data collection standards for race, ethnicity, sex, primary language, and disability status. [cited 3 May 2022]. Available: <https://minorityhealth.hhs.gov/data-collection-standards-race-ethnicity-sex-primary-language-and-disability-status>

Reference for framework of SES data collection and analysis: Braveman PA, Cubbin C, Egerter S, Chideya S, Marchi KS, Metzler M, et al. Socioeconomic status in health research: one size does not fit all. JAMA. 2005;294: 2879–2888. doi:10.1001/jama.294.22.2879

Reference on development and domain description of SDOH screening tool: Billioux A, Verlander K, Anthony S, Alley D. Standardized screening for health-related social needs in clinical settings: The Accountable Health Communities Screening Tool. NAM Perspectives. 2017. doi:10.31478/201705b

REDCap citation: Harris PA, Taylor R, Thielke R, Payne J, Gonzalez N, Conde JG. Research electronic data capture (REDCap)—A metadata-driven methodology and workflow process for providing translational research informatics support. Journal of Biomedical Informatics. 2009;42: 377–381. doi:10.1016/j.jbi.2008.08.010

**Reference removed:**

Erroneous remnant of a SDOH assessment tool that was considered but not incorporated into the protocol: Gottlieb L, Hessler D, Long D, Amaya A, Adler N. A randomized trial on screening for social determinants of health: the iScreen study. Pediatrics. 2014 Dec;134(6):e1611-1618.

Reviewers' comments:

Reviewer's Responses to Questions

**Comments to the Author**

1. Does the manuscript provide a valid rationale for the proposed study, with clearly identified and justified research questions?

The research question outlined is expected to address a valid academic problem or topic and contribute to the base of knowledge in the field.

Reviewer #1: Yes

Reviewer #2: Yes

2. Is the protocol technically sound and planned in a manner that will lead to a meaningful outcome and allow testing the stated hypotheses?

The manuscript should describe the methods in sufficient detail to prevent undisclosed flexibility in the experimental procedure or analysis pipeline, including sufficient outcome-neutral conditions (e.g. necessary controls, absence of floor or ceiling effects) to test the proposed hypotheses and a statistical power analysis where applicable. As there may be aspects of the methodology and analysis which can only be refined once the work is undertaken, authors should outline potential assumptions and explicitly describe what aspects of the proposed analyses, if any, are exploratory.

Reviewer #1: Yes

Reviewer #2: Yes

3. Is the methodology feasible and described in sufficient detail to allow the work to be replicable?


Descriptions of methods and materials in the protocol should be reported in sufficient detail for another researcher to reproduce all experiments and analyses. The protocol should describe the appropriate controls, sample size calculations, and replication needed to ensure that the data are robust and reproducible.

Reviewer #1: Yes

Reviewer #2: Yes

4. Have the authors described where all data underlying the findings will be made available when the study is complete?

The [PLOS Data policy](https://nam04.safelinks.protection.outlook.com/?url=https%3A%2F%2Furldefense.com%2Fv3%2F__https%3A%2F%2Fjournals.plos.org%2Fplosone%2Fs%2Fmaterials-and-software-sharing__%3B!!OToaGQ!rQMlusJBBcqeJ1PSZVlq_mjiSDK4aFCHvyNOOyQ2usv1fMmOqHfFR1Wej2IaoonN_etN33FjDv4fkz25BQ%24&data=05%7C02%7Cbriajh%40vt.edu%7C41f5a54f31e2498b9f9b08dc3ca2d99a%7C6095688410ad40fa863d4f32c1e3a37a%7C0%7C0%7C638451917477448854%7CUnknown%7CTWFpbGZsb3d8eyJWIjoiMC4wLjAwMDAiLCJQIjoiV2luMzIiLCJBTiI6Ik1haWwiLCJXVCI6Mn0%3D%7C0%7C%7C%7C&sdata=QkEcUh%2BrzupUz4jraD8haX3okDiIREX%2Fifb36q5ZcPA%3D&reserved=0) requires authors to make all data underlying the findings described in their manuscript fully available without restriction, with rare exception, at the time of publication. The data should be provided as part of the manuscript or its supporting information, or deposited to a public repository. For example, in addition to summary statistics, the data points behind means, medians and variance measures should be available. If there are restrictions on publicly sharing data—e.g. participant privacy or use of data from a third party—those must be specified.

Reviewer #1: Yes

Reviewer #2: Yes

5. Is the manuscript presented in an intelligible fashion and written in standard English?

PLOS ONE does not copyedit accepted manuscripts, so the language in submitted articles must be clear, correct, and unambiguous. Any typographical or grammatical errors should be corrected at revision, so please note any specific errors here.

Reviewer #1: Yes

Reviewer #2: Yes

6. Review Comments to the Author

Please use the space provided to explain your answers to the questions above and, if applicable, provide comments about issues authors must address before this protocol can be accepted for publication. You may also include additional comments for the author, including concerns about research or publication ethics.

You may also provide optional suggestions and comments to authors that they might find helpful in planning their study.

(Please upload your review as an attachment if it exceeds 20,000 characters)

Reviewer #1: The is very useful and comprehensive study proposal with a well defined study design. The tools that will be used for data collection are well explained with the respect to the research questions.
However the authors should mention why data analysis plan for establishing the SES classification should be based on the work done by Sheffer et al (2012), reference 25

Great point. We have expanded on why we chose this SES classification for this population in the “demographic and clinical data section”. As illustrated in the manuscript text, we chose the composite index of SES using the method described by Shaffer et al (2012) due to its granular, family-level factors of income categories based on US Census Bureau median household income and discrete levels of educational attainment. Income, education level, and occupation are family-level measures commonly used in SES indices in the literature; however, Shaffer's approach incorporate the practical benefit of discrete categories to protect patient privacy (as opposed to direct income reporting) and allow for reconstruction of SES indices if interim analysis illustrates that the division resulted in a bias split of the data. Broader, population level measures of SES were considered, such as the Area Deprivation Index (ADI); however, as our study is not a large-scale epidemiological study, we chose to focus on SES status driven by family- level data. Lastly, Braveman et al (2005) highlighted the importance of analyzing individual socioeconomic factors in analysis when also using composite SES scores, as such, we are collecting other variables known to impact SES and will systematically account for this during analysis. Qualitative interviews will expand on subjective definitions of SES and may identify key unmeasured factors.

Reviewer #2: It is a very well-designed protocol and I agree that the mixed methods design will enhance the results of the study.
I don't know how the primary care is given in the 2 centers or if the patient's caregivers may consult a family practice or pediatrician before going to the emergency room.
Suppose the patient may seek for consulting early, but the primary-care physician delays the access to specialized care. In that case, it may be a confounding factor that is not evaluated in the protocol. So, maybe, besides the perforation rate it would be helpful to add another variable: the "time since symptoms began to first medical consulting".
Another factor that may influence perforation is an in-hospital delay of surgery. Suppose that, for any reason, the patient is not operated on promptly and the appendix may rupture. That’s not on the family experiences.
Other than that, it is a very good protocol, and I will be looking to find the results.

We agree with this suggestion, and it will be important to adjust for these factors in the analysis. We accounted for this in the protocol, although not previously articulated in the manuscript, and are collecting several time-related variables, including family-reported variables (i.e., time onset of symptom, time to initial healthcare access point) and EMR-reported variables (i.e., ED arrival time, time of surgery, discharge time). These variables will enable our analysis team to adjust for other factors that drive delays in care. We are also collecting the contexts of “initial healthcare access point(s)” before ED presentation (pediatrician, outside emergency room, urgent care). We have further detailed the collection of these variables in our methods section, lines 233-235.

7. PLOS authors have the option to publish the peer review history of their article ([what does this mean?](https://nam04.safelinks.protection.outlook.com/?url=https%3A%2F%2Furldefense.com%2Fv3%2F__https%3A%2F%2Fjournals.plos.org%2Fplosone%2Fs%2Feditorial-and-peer-review-process*loc-peer-review-history__%3BIw!!OToaGQ!rQMlusJBBcqeJ1PSZVlq_mjiSDK4aFCHvyNOOyQ2usv1fMmOqHfFR1Wej2IaoonN_etN33FjDv4Y3kNu9w%24&data=05%7C02%7Cbriajh%40vt.edu%7C41f5a54f31e2498b9f9b08dc3ca2d99a%7C6095688410ad40fa863d4f32c1e3a37a%7C0%7C0%7C638451917477455349%7CUnknown%7CTWFpbGZsb3d8eyJWIjoiMC4wLjAwMDAiLCJQIjoiV2luMzIiLCJBTiI6Ik1haWwiLCJXVCI6Mn0%3D%7C0%7C%7C%7C&sdata=3vtTBltbVLFN6g0m%2FDI4xSwfRp7kg7IPvRQ8L7fjW6E%3D&reserved=0)). If published, this will include your full peer review and any attached files.


If you choose “no”, your identity will remain anonymous but your review may still be made public.


**Do you want your identity to be public for this peer review?** For information about this choice, including consent withdrawal, please see our [Privacy Policy](https://nam04.safelinks.protection.outlook.com/?url=https%3A%2F%2Furldefense.com%2Fv3%2F__https%3A%2F%2Fwww.plos.org%2Fprivacy-policy__%3B!!OToaGQ!rQMlusJBBcqeJ1PSZVlq_mjiSDK4aFCHvyNOOyQ2usv1fMmOqHfFR1Wej2IaoonN_etN33FjDv6zNiaSVA%24&data=05%7C02%7Cbriajh%40vt.edu%7C41f5a54f31e2498b9f9b08dc3ca2d99a%7C6095688410ad40fa863d4f32c1e3a37a%7C0%7C0%7C638451917477461704%7CUnknown%7CTWFpbGZsb3d8eyJWIjoiMC4wLjAwMDAiLCJQIjoiV2luMzIiLCJBTiI6Ik1haWwiLCJXVCI6Mn0%3D%7C0%7C%7C%7C&sdata=LjULPDQnp5DdmKPaqwGWQC9KOxvNpjlcA2T3tCflHhw%3D&reserved=0).

Reviewer #1: No

Reviewer #2: **Yes:** Eduardo Bracho-Blanchet
